# In Silico Evaluation of Natural Compounds for an Acidic Extracellular Environment in Human Breast Cancer

**DOI:** 10.3390/cells10102673

**Published:** 2021-10-06

**Authors:** YoungJoon Park, Jaekwang Jeong, Shin Seong, Wonnam Kim

**Affiliations:** 1 Cnh Center for Cancer Research, Cnh Corporation, Gangnam-gu, Seoul 06154, Korea; yjparkcb@cnhgroup.kr; 2 Section of Endocrinology and Metabolism, Department of Internal Medicine, Yale University School of Medicine, New Haven, CT 06519, USA; jaekwang.jeong@yale.edu; 3 Soram Korean Medicine Hospital, Gangnam-gu, Seoul 06154, Korea; ss9335@soramhospital.kr

**Keywords:** acidic tumor microenvironment, breast cancer, natural compound, Bruceine D

## Abstract

The survival rates for breast cancer (BC) have improved in recent years, but resistance, metastasis, and recurrence still remain major therapeutic challenges for BC. The acidic tumor microenvironment (TME) has attracted attention because of its association with tumorigenesis, metastasis, drug resistance, and immune surveillance. In this study, we evaluated natural compounds from traditional herbal medicine used to treat cancer that selectively target genes regulated by extracellular acidosis. We integrated four transcriptomic data including BC prognostic data from The Cancer Genome Atlas database, gene expression profiles of MCF-7 cells treated with 102 natural compounds, patterns of gene profiles by acidic condition, and single-cell RNA-sequencing from BC patient samples. Bruceine D (BD) was predicted as having the highest therapeutic potential, having an information gain (IG) score of 0.24, to regulate reprogrammed genes driven by acidosis affecting the survival of BC patients. BD showed the highest IG on EMT (IG score: 0.11) and invasion (IG score: 0.1) compared to the other phenotypes with the CancerSEA database. BD also demonstrated therapeutic potential by interfering with the tumor cell–TME interactions by reducing the amyloid beta precursor protein and CD44 expression. Therefore, BD is a potential candidate to target the acidic TME induced metastatic process in BC.

## 1. Introduction

In 2020, breast cancer (BC) was the most diagnosed cancer at 24.5% and was the leading cause of death at 15.5% among women with cancer [1]. Advances in surgical techniques, radiation therapy, and systemic therapies for BC contributed to an increase in the 5-year relative survival rate [2,3]. Although BC mortality has continued to improve over the past decades, radiation resistance, drug resistance, and metastatic recurrence still remain major therapeutic challenges for BC [4,5,6]. Solid tumors produce and export excessive levels of lactic acid due to reprogrammed cancer cell metabolism, and when combined with poor vascular perfusion, it eventually leads to an acidic extracellular pH (pH_e_) microenvironment [7,8]. The adaptation of cancer cells to acidic pH_e_ within the tumor microenvironment (TME) is an important factor in increasing tumor aggressiveness such as invasion and metastasis [9,10,11,12]. In acidic regions, tumor cells perform niche engineering that results in extracellular matrix degradation, normal cell death, and local invasiveness [13,14]. Tumors with higher metastatic potential showed lower pH values, dispersedly, in peritumoral regions [15]. Additionally, distant metastasis was associated with enhanced tumor acidity [16]. In BC, exposure to acidic pH_e_ increased cell migration and drug resistance in MCF-7 cells [17]. Drug-resistant MCF-7 cells showed lowered pH_e_ compared to parent BC cells [18]. Comparison between metastatic 4T1 and less metastatic TUBO cells showed that pH_e_ correlated with distant lung metastasis [19].

**Figure 1:**
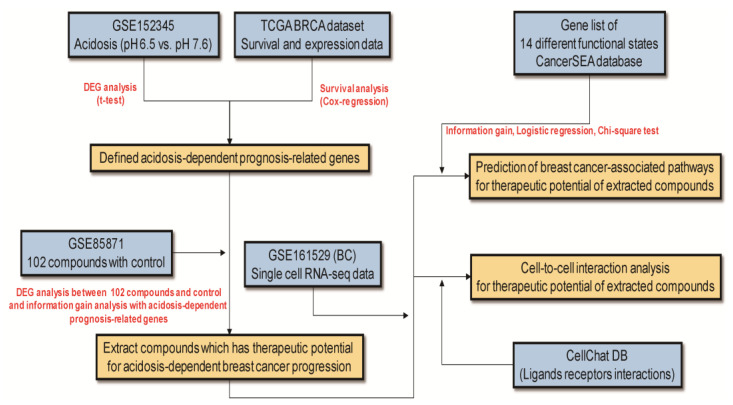
Workflow.

Traditional herbal medicine has long been used to treat cancer, as some herbs contain natural compounds with anticancer effects that target proliferation, angiogenesis, metastasis, and apoptosis [20]. Natural compounds, such as epigallocatechin gallate, curcumin, berberine, artemisinins, ginsenosides, and silibinin, have been reported to regulate autophagy, drug resistance, immunity balance, and chemosensitization in vitro and in vivo [20]. In this study, we analyzed the transcriptomic expression patterns of genes by acidic condition, the BC prognostic data from The Cancer Genome Atlas (TCGA) database, and the gene expression profiles of MCF-7 cells treated with 102 natural compounds from herbal medicine (Figure 1). Moreover, we predicted potential cancer-associated pathways that could be improved by compounds with therapeutic potential for acidic pH_e_. Finally, we investigated how compounds would affect receptor–ligand interactions in the TME using single-cell sequencing of BC patient samples (Figure 1).

## 2. Materials and Methods

### 2.1. Data Collection

We downloaded GSE152345 transcriptomic (Illumina HiSeq platform) and survival data of a BC database from Xena TCGA (https://xenabrowser.net/hub/, accessed on 27 May 2021). The GSE152345 with a previous study contains whole gene expression levels through high-throughput sequencing with BGISEQ-500 between pH 6.5 and pH 7.6 conditions of the MCF-7 cell line [21]. In addition, we downloaded GSE85871, which contains gene expression profiles of the MCF-7 cell line microarray (Affymetrix human genome U133A 2.0 array) treated with 102 traditional Chinese medicine-related compounds [22]. The gene list with 14 different functional states of tumor cells was downloaded from CancerSEA (http://biocc.hrbmu.edu.cn/CancerSEA/home.jsp, accessed on 10 August 2021) [23]. Finally, we downloaded GSE161529, which contains single-cell sequencing data of tissues from each ER^+^, HER2^+^, and triple-negative (TN) BC patient [24].

### 2.2. Data Processing

GSE152345, which contains expression profiles between acidic (pH 6.5) and normal (pH 7.6) conditions within the MCF-7 BC luminal type, was normalized with the DESeq2 package of R (https://bioconductor.org/packages/release/bioc/html/DESeq2.html, accessed on 24 May 2021) and transformed log2(x + 1) [25]. GSE85871 was downloaded by the GEOquery package of R, and duplicated gene symbols were matched according to probe IDs.

### 2.3. Acidosis-Dependent Prognosis-Related Genes

To analyze acidosis-dependent prognosis-related genes, we performed survival analysis with Cox-regression and a t-test for extracting differential expressed genes with TCGA BC and GSE152345 data, respectively. After that, genes that were commonly significant in both analyses were classified into 4 types according to hazard ratio and fold change. The 4 types were risk effect to prognosis and up-regulated in acidic condition (RU), protective effect to prognosis and up-regulated in acidic condition (PU), risk effect to prognosis and down-regulated in acidic condition (RD), and protective effect to prognosis and down-regulated in acidic condition (PD).

### 2.4. Selection Method to Identify Compounds with Therapeutic Potential against Acidosis-Dependent Prognosis-Related Genes

Information gain (IG) was considered as an evaluation parameter to select compounds with therapeutic potential in association with expression patterns of the acidosis-dependent prognosis-related genes. According to the fold change value 0, among the up- or down-regulation genes, the proportion of RU or PD was calculated based on Shannon’s entropy, and the IG score was calculated.

Let us assume probability (*P*) and *i* contain a number of RU (*ru*) and PD (*pd*); therefore, Shannon’s entropy was calculated as follows:

$$Entropy=-\underset{i=\left\{ru,\;pd\right\}}{\sum }P\left(i\right)\cdot log_{2}P\left(i\right)$$

Let us assume T as the population of targets such as RU or PD before splitting according to the fold change 0, and *s* contains up-regulated (*u*) and down-regulated (*d*). The IG of each compound was calculated as follows:

$$IG\left(T,\;X\right)=Entropy\left(T\right)-\underset{s=\left\{u,d\right\}}{\sum }\frac{s}{T}\cdot Entropy\left(s\right)$$

### 2.5. Evaluation of Compound Effect on 14 Different Functional States from CancerSEA Database

We performed logistic regression and calculated IG values between those up- or down-regulated by the compound treated group and directions such as positive or negative against 14 different functional states from the CancerSEA database, including angiogenesis, apoptosis, DNA damage, DNA repair, epithelial mesenchymal transition (EMT), invasion, differentiation, proliferation, cell cycle, metastasis, hypoxia, stemness, inflammation, and quiescence. The IG was obtained by the calculation formula described above.

Let us assume probability (*P*) and *i* contain a number of positives (*p*) and negatives correlated (*n*) with each 14 different functional states; the Shannon’s entropy was calculated as follows:

$$Entropy=-\underset{i=\left\{p,\;n\right\}}{\sum }P\left(i\right)\cdot log_{2}P\left(i\right)$$

Let us assume the *T* as a population of the targets such as RU or PD before splitting according to the fold change 0, where *s* contains up-regulated (*u*) and down-regulated (*d*). The IG of each compound is calculated as follows:

$$IG\left(T,\;X\right)=Entropy\left(T\right)-\underset{s=\left\{u,d\right\}}{\sum }\frac{s}{T}\cdot Entropy\left(s\right)$$

The logistic regression was performed with directions for each 14 different functional states as a dependent variable and up- and down-regulated in compound treated states as an independent variable.

### 2.6. Single-Cell Sequencing Analysis

We downloaded 3 single-cell RNA sequencing (scRNA-seq) data of ER^+^ (GSM4909299, GSM4909307, and GSM4909315), HER2^+^ (GSM4909289, GSM4909290, and GSM4909294), and TN (GSM4909281, GSM4909282, and GSM4909288), each from GSE161529. Tumor cells in each subtype including ER^+^, HER2^+^, and TN were extracted according to their specific markers: *KRT5*^−^, *KRT18*^+^, and *ESR1*^+^ (ER^+^); *KRT5*^−^, *KRT18*^+^, and *ERBB2*^+^ (HER2^+^); and *KRT5*^+^ and *KRT18*^+^ (TN). After extracting tumor cells from the single-cell population, we calculated the percentage of cells expressing each marker within the extracted tumor cells. All of the processes were performed by Seurat (https://satijalab.org/seurat/, accessed on 3 August 2021) [26].

### 2.7. Cell-to-Cell Interaction Analysis with scRNA-seq

We downloaded scRNA-seq data from normal (GSM4909262) samples, and ER^+^ tumor (GSM4909313) paired samples were downloaded from GSE161529. Clustering and annotation of cell types of normal and ER^+^ tumors were performed by Seurat [26] with default options, and cell-to-cell communication analysis was conducted with the CellChat (http://www.cellchat.org/, accessed on 19 August 2021) package of R [27]. The interaction database was retrieved from CellChatDB, which contains a total of 2,021 molecular interactions including paracrine/autocrine (60%), ECM-related (21%), and cell–cell contact (19%) interactions [27].

### 2.8. Statistical Analysis

We performed t-test, Cox-regression, and logistic regression analyses in this study. The threshold of the *p*-value is < 0.05 with a t-test and Cox-regression without adjusting for multiple comparisons. With logistic regression, we applied Bonferroni corrections for adjusting multiple comparisons. All of the statistical analyses were performed by Rstudio (Version 1.41106) and Python (Version 3.9).

## 3. Results

### 3.1. Identification of Acidosis-Dependent Prognosis-Related Genes

**Figure 2:**
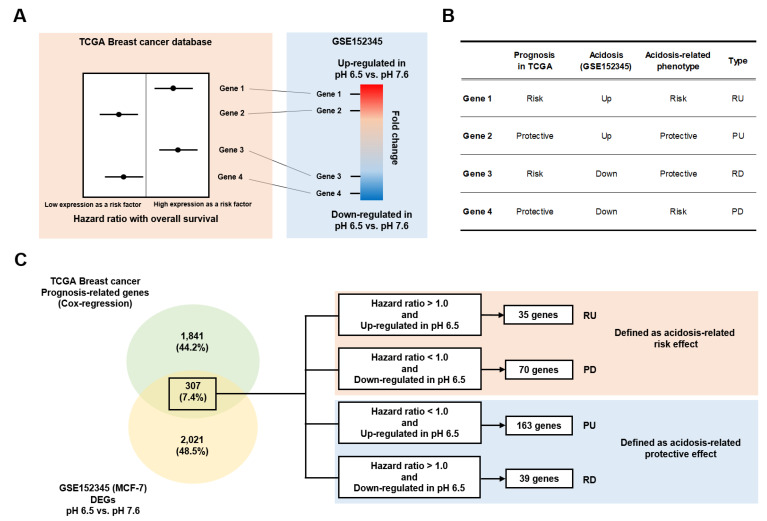
Conception and results of extraction of acidosis-dependent prognostic genes. (**A**) Conception of linkage between prognostic and acidosis relationship, (**B**) defined types for acidosis-dependent prognostic genes, and (**C**) classification of the genes according to the conception and definition.

To identify acidosis-dependent prognosis-related genes, a Cox-regression and t-test were performed between acidic (pH 6.5) and normal (pH 7.6) conditions with GSE152345 based on whole gene expression for the overall survival from TCGA, respectively (Figure 2A, Tables S1 and S2). According to the hazard ratio and fold change, we classified the data into four types such as RU, PU, RD, and PD (Figure 2B). As a result, 2148 (Table S1) and 2328 (Table S2) significant genes were extracted with the Cox-regression for extracting prognosis-related genes and the t-test to identify differential expressed genes (DEGs), respectively (Figure 2C). Among these significant genes, 307 genes were significant in both statistical analyses (Figure 2C and Table S3). According to the hazard ratio and fold change, 35, 70, 163, and 39 genes were defined as RU, PD, PU, and RD, respectively (Figure 2C).

### 3.2. Evaluation of Therapeutic Compounds against an Acidosis-Dependent Manner in BC

**Figure 3:**
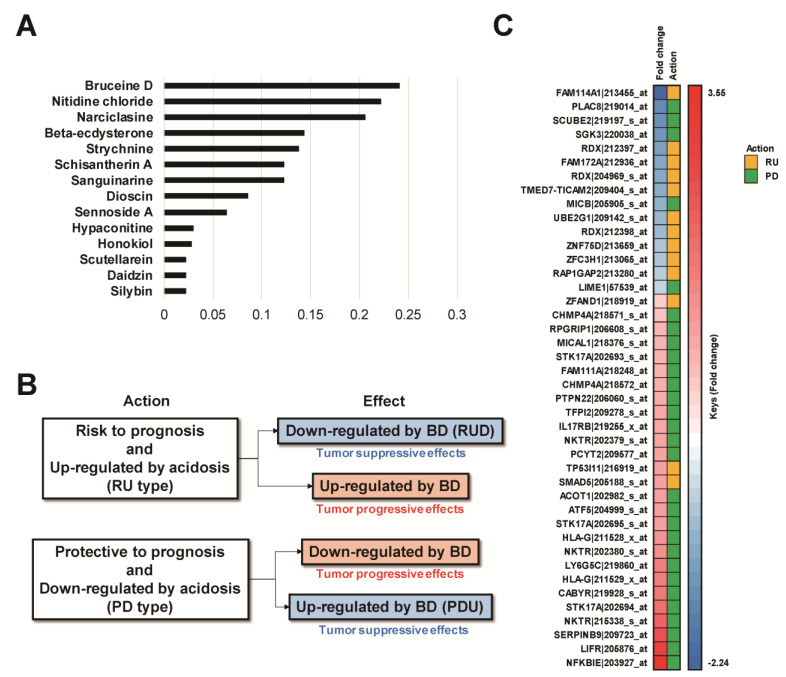
Evaluation of therapeutic potential of the 102 compounds for acidosis-dependent prognostic genes. (**A**) Evaluation with IG score, (**B**) defined effect of BD on RU and PD genes, and (**C**) distribution and patterns of the genes which were affected by BD within acidosis-dependent prognostic genes.

For this study, 102 traditional herbal medicine-related compounds were evaluated against acidic conditions. We focused on RU- or PD-type genes that cause an acidosis-related risk effect by up-regulating risk genes or down-regulating protective genes, respectively. Among the 102 compounds, Bruceine D (BD) had the highest IG score (0.24) with RU and PD (Figure 3A). Among the 27 up-regulated probes in the BD-treated group, 24 probes were PD type, which indicates a better prognosis and is down-regulated by acidic (pH 6.5) conditions (PDU) (Figure 3B,C). In addition, among the 15 down-regulated probes in the BD-treated group, 10 probes were RU type, which indicates a poor prognosis and is up-regulated by acidic (pH 6.5) conditions (RUD) (Figure 3B,C).

### 3.3. Effect of BD on 14 Different Cancer-Related Functional States with the CancerSEA Database

**Figure 4:**
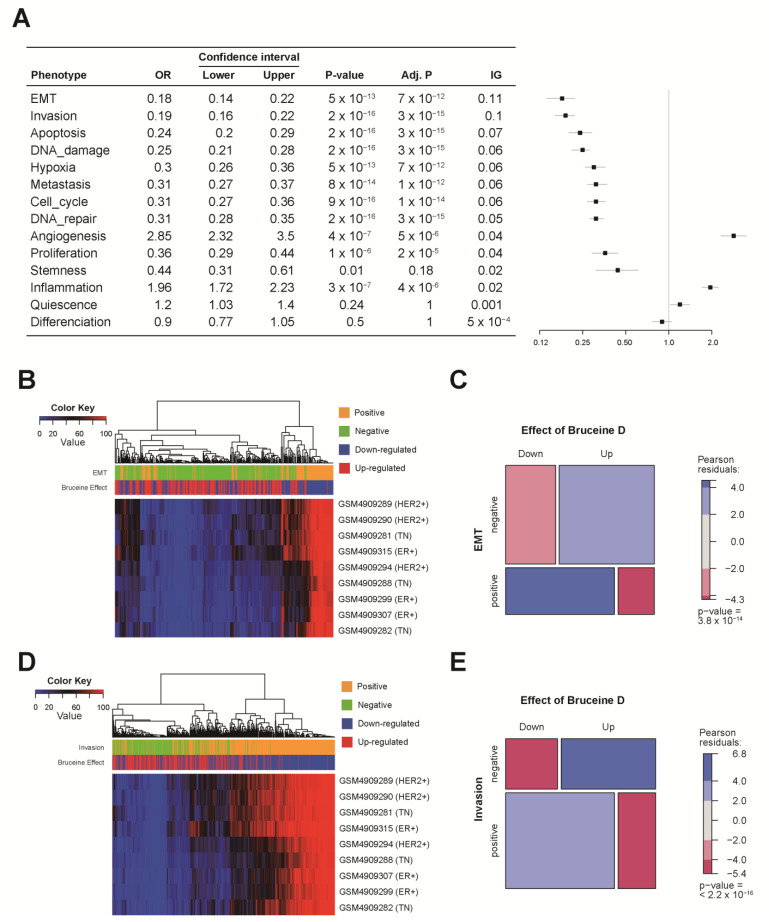
Evaluations with 14 cancer cell-associated functional states. (**A**) Forest plot and table with logistic regression and IG score. (**B**) Patterns of EMT associated DEGs with BD genes, and percentages of positive cells within segmented tumor cells from scRNA-seq data. (**C**) Mosaic plot with Chi-square test and Pearson residuals between EMT and DEGs with BD genes. (**D**) Patterns of invasion-associated DEGs with BD genes and percentages of positive cells within segmented tumor cells from scRNA-seq data. (**E**) Mosaic plot with Chi-square test and Pearson residuals between invasion and DEGs with BD genes.

To evaluate the effect of BD on tumor cell-related functional states, we downloaded genes and directions with each of the 14 different BC-related functional states including angiogenesis, apoptosis, DNA damage, DNA repair, EMT, invasion, differentiation, proliferation, cell cycle, metastasis, hypoxia, stemness, inflammation, and quiescence (Figure 4). In addition, with the nine total patients, including ER^+^, HER2^+^, and TN, cancer cells from a single cell population were segmented (Figures S1–S3), and percentages of positive cells from each of the 14 different tumorigenesis-related functional states were calculated (Figure 4B,D and Figure S4). As a result, EMT and invasion showed higher IG values > 0.1 compared to the other phenotypes (Figure 4A). Interestingly, the genes that positively correlated with EMT and invasion were highly expressed in the cancer cell populations (Figure 4B,D). Our data showed that BD up-regulated genes had negative correlations with EMT, whereas down-regulated genes had positive correlations with invasion(Figure 4C,E).

### 3.4. Effect of BD on Cell-to-Cell Interaction Analysis with scRNA Resolution

**Figure 5:**
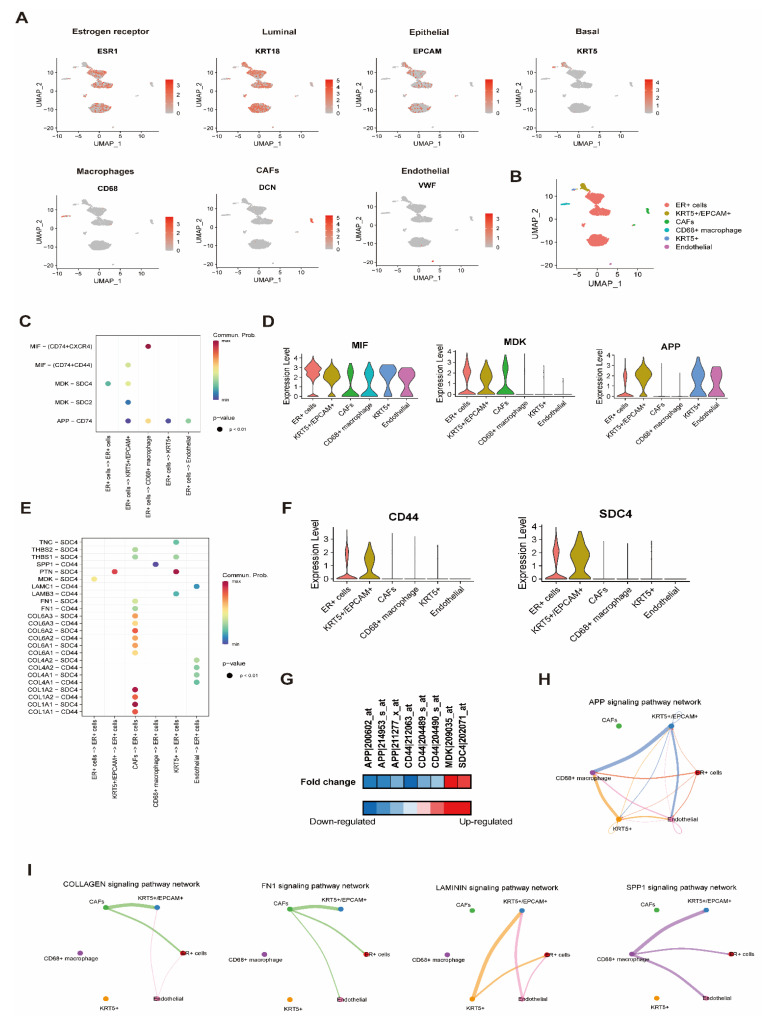
Cell-to-cell interaction analysis with scRNA-seq data from the ER^+^ patient (GSM4909313). (**A**) Dim plots with markers of each cell type. (**B**) Dim plot of classified cell types according to the markers of each cell type. (**C**) Dot plot of ligand–receptor interactions based on ligands expressed in ER^+^ cells. (**D**) Violin plots of three ligands which were expressed in ER^+^ cells. (**E**) Dot plot of ligand–receptor interactions based on receptors expressed in ER^+^ cells. (**F**) Violin plots of two receptors which were expressed in ER^+^ cells. (**G**) Heatmap plot of significantly down- or up-regulated genes by BD. (**H**) Ligand–receptor interaction network based on the APP signaling pathway as a ligand in ER^+^ cells, and (**I**) ligand–receptor interaction network based on four pathways as ligands on the CD44 receptor in ER^+^ cells.

At the single-cell level, we investigated the effect of BD on ligand–receptor interactions within the TME. The scRNA-seq dataset of the normal and ER^+^ paired sample was used for cell-to-cell interaction analysis. As a result, the ER^+^ cancer sample was clustered with ER^+^ tumor cells (*ESR1*^+^, *KRT18*^+^, and *KRT5*^−^), *KRT5*^+^/*EPCAM*^+^ double-positive cells, cancer-associated fibroblasts (CAFs) (*DCN*^+^), macrophages (*CD68*^+^), *KRT5*^+^/*EPCAM*^−^ cells, and endothelial cells (*VWF*^+^) (Figure 5A,B). In addition, the normal sample was clustered with epithelial cells (*KRT18*^+^ and *EPCAM*^+^), *KRT5*^+^ cells (*KRT5*^+^), and fibroblasts (*DCN*^+^) (Figure S5A,B). Since DEGs analysis for BD was performed in MCF-7 cells, we analyzed the ligand–receptor relationship based on ER^+^ cells (Figure 5C,E). The three ligands including *MIF* (encodes macrophage migration inhibitory factor), *MDK* (encodes midkine), and *APP* (encodes amyloid precursor protein) were expressed in ER+ cells (Figure 5D) and interacted with receptors including *CD74*, *CXCR4*, *CD44*, *SDC4* (encodes syndecan 4), and *SDC2* (encodes syndecan 2) expressed in various cell types (Figure 5C and Figure S6A). The two receptors including *SDC4* and *CD44* were expressed in ER^+^ cells (Figure 5E,F) and interacted with ligands such as the collagen family (*COL1A1*, *COL1A2*, *COL4A1*, *COL4A2*, *COL6A1*, *COL6A2*, and *COL6A3*), *FN1* (Fibronectin 1), the laminin family (*LAMC1* and *LAMB3*), *MDK*, *PTN* (Pleiotrophin), *SPP1* (Osteopontin), *THBS1* (Thrombospondin 1), *THBS2* (Thrombospondin 2), and *TNC* (Tenascin C) expressed in various cell types (Figure 5E and Figure S6B). Among these ligand–receptor relationships, *APP* and *CD44* were significantly down-regulated by BD in the MCF-7 cell line (Figure 5G). As a ligand, *APP* was down-regulated by BD and interacted with various cell types in ER^+^ TME via *CD74* (Figure 5C,H). In addition, BD down-regulated *CD44,* which interacted with several collagen types such as FN1, laminins, and SPP1 (Figure 5E,I). These findings were not detected in normal scRNA-seq data (Figure S5).

## 4. Discussion

Solid tumors adapt and survive in acidic TME through pH-regulating proteins such as the sodium/proton exchanger 1 (NHE1), sodium bicarbonate cotransporter (NBC), monocarboxylate transporters (MCT), and carbonic anhydrase [28,29]. The acidic TME has received attention due to its association with tumor development, progression, metastasis, drug resistance, and escape from immune surveillance [28,30]. The advances in modern technology have increased our understanding of the pharmacology and molecular mechanisms of traditional herbal medicine and its active compounds [31]. Natural compounds exert anti-cancer effects on the TME that consists of tumor cells, stromal cells, immune cells, and non-cellular components such as collagen and fibronectin either directly or indirectly [20,32]. In this study, we used a computational approach to evaluate 102 major TCM-related natural compounds for an acidic pH_e_ environment in human BC.

After evaluation between 102 compounds and BC cells under an acidic pH_e_, BD was suggested as the candidate with the most potential to treat BC against an experimental acidic condition with the highest IG value (IG value: 0.24) (Figure 3A and Table S5). BD is a major active quassinoid in *Brucea javanica* (L.) Merr., which has anti-cancer properties such as anti-proliferative and pro-apoptotic effects via c-Jun N-terminal kinase (JNK), mitogen-activated protein kinases (MAPK), phosphatidylinositol 3-kinase (PI3K)/protein kinase B (AKT)/mammalian target of Rapamycin (mTOR), and canonical Wnt signaling pathways against many cancers including BC [33,34]. Our data showed that 10 probes defined as RUD and 24 probes defined as PDU in BC cells were potential therapeutic targets of BD. Moreover, the number of genes defined by PDUs was higher than those defined by RUDs (Figure 3C). This suggests that BD exhibits a pattern to prevent cancer progression by up-regulating the protective genes (*NFKBIE*, *LIFR*, *SERPINB9*, etc.), which were down-regulated due to acidification, rather than down-regulating the risk genes (*FAM114A1*, *PLAC8*, *SCUBE2*, etc.), which were up-regulated due to acidification.

**Figure 6:**
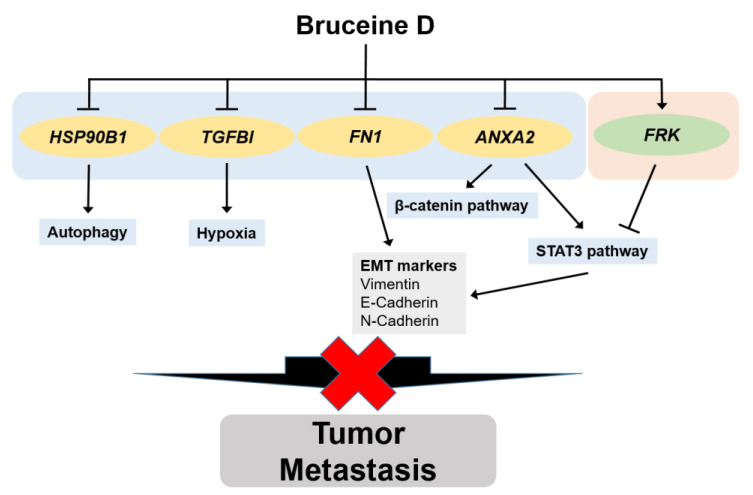
Schematic representation for the inhibitory effect of BD on tumor metastasis.

We used the CancerSEA database aimed at identifying the correlation between BD and 14 functional states. The DEGs for BD were evaluated by the IG method, with the highest discrimination powers being against EMT and invasion with an IG score of >0.1 (Figure 4A). Our segmented scRNA-seq data originating from ER^+^, HER2^+^, and TN BC further supported that higher proportions were seen in cell populations that express positive-related genes compared to negative-related genes against EMT or invasion (Figure 4B,D). Prior studies have proposed the regulatory effect of BD on EMT, invasion, and apoptosis. BD inhibited STAT3 (Signal Transducer and Activator of Transcription 3) activation that attenuated the cell proliferation, migration, invasion, and stem cell-like properties of osteosarcoma cells [35]. BD dose-dependently increased E-cadherin, whereas BD decreased vimentin and β-catenin expression that resulted in the reduced migration and invasion abilities of MDA-MB-231 cells [36]. BD increased oxidative stress and inhibited the PI3K/Akt signaling pathway, inducing apoptosis in human pancreatic cancer cells [37]. Interestingly, we found that BD regulated genes with positive (*ANXA2*, *HSP90B1*, *TGFBI*, *FN1*) and negative (*FRK*) correlation with EMT, invasion, and metastasis. Overexpressed *ANXA2* (Annexin A2) exhibits poor prognosis and correlates positively with invasion and metastasis in BC [38]. Annexin A2 interacts with STAT3 and mediates EGF (Epidermal growth factor) induced EMT [38]. *ANXA2* knockdown suppressed β-catenin expression and inhibited EMT and invasion in ovarian cancer cells [39]. GRP94 (Glucose-regulated protein 94, *HSP90B1*) expression highly correlates with brain metastatic BC, and autophagy mediated the adaption and survival at metastatic sites [40]. *FN1* (Fibronectin 1) is upregulated in various tumors and mediates cell proliferation and migration [41]. *FN1* knockdown showed decreased cell migration and invasion by modulating EMT-related proteins such as E-cadherin, N-cadherin, and vimentin in MCF-7 cells [42]. In silico analysis showed that *TGFBI* (Transforming growth factor beta induced protein) is associated with poor prognosis and aggressive BC subtypes [43]. An in vivo study showed that *TGFBI* affected hypoxia and metastasis in BC [43]. Previous studies have shown the tumor suppressive role of *FRK* (Fyn-related kinase) by inhibiting cell proliferation, PTEN (Phosphatase and tensin homolog) degradation, and EGF receptor signaling in BC [44,45,46]. Overexpression of *FRK* inhibited STAT3 activation, which suppressed the EMT process and cell migration in BC cells [47]. BD treatment may induce *ANXA2*, *TGFI*, *FN1*, *HSP90B1* down-regulation, and *FRK* up-regulation to inhibit tumor metastasis by regulating autophagy, hypoxia, β-catenin, and STAT3 signaling pathways (Figure 6).

We next determined whether BD interferes with the tumor cell–TME interactions. The cellular heterogeneity in the TME includes immune cells, fibroblasts, and stromal cells [48]. For this reason, we performed cell-to-cell interaction analysis with single-cell resolution. As a result, among the various ligand–receptor relationships in ER^+^ TME, BD decreased *APP* and *CD44* expression (Figure 5G). The *APP* as a ligand interacted with *CD74,* which was mainly expressed in *CD68*^+^ macrophages in the cell-to-cell interaction analysis (Figure 5C and Figure S6A). *APP* encodes an amyloid beta precursor protein and is strongly linked to Alzheimer’s disease [49]. Interaction between APP and CD74 reduces the production of beta amyloid in Alzheimer’s disease [50]. Unfortunately, there is no evidence of a connection between APP and CD74 in cancer. However, *APP* is up-regulated in BC cells and tissues, which promotes tumor formation and progression [51,52]. *CD44* is a member of the cell adhesion molecules that have been proposed as having conflicting functions, i.e., either being tumor-promoting or tumor-suppressing in BC [53]. *CD44* promotes cancer cell migration and invasion by directly interacting with MMP-9, which degrades collagens [54,55]. CD44 stimulates multidrug resistance protein (P-glycoprotein) expression and leads to chemoresistance in BC cells [56]. Our results demonstrated that *CD44* within ER^+^ tumor cells interacted with *SPP1*, also known as osteopontin (OPN) with macrophages (Figure 5E and Figure S6B). A previous study revealed that the tumor-associated macrophages interacted with CD44^+^ cancer stem cells and enhanced tumorigenesis via the OPN/CD44 axis [57]. Additionally, OPN induced radiation resistance by activating the CD44 signaling pathway in glioma [58].

In summary, we used in silico methodology to select the most potent compound to target genes characterized by acidic pH_e_ that influence the survival of BC patients. Among the 102 natural compounds, BD showed the highest potential to regulate reprogrammed genes driven by acidosis, affecting the prognosis of BC. BD down-regulated genes that positively correlate with EMT and invasion, while it up-regulated genes that negatively correlate with EMT and invasion. BD showed therapeutic potential by changing the TME condition by reducing *APP* and *CD44* expression. Taken together, BD may be an effective natural compound for treating BC metastasis driven by extracellular acidity. More research is needed to fully understand the mechanism of the action of BD in acidic TME.

## Data Availability

The datasets presented in this study are available from the corresponding author upon request.

## Supplementary Materials

The following are available online at https://www.mdpi.com/article/10.3390/cells10102673/s1, Figure S1: Segmentation of ER+ breast cancer cells from single cell data with patients, Figure S2: Segmentation of HER+ breast cancer cells from single cell data with patients, Figure S3: Segmentation of TN breast cancer cells from single cell data with patients, Figure S4: Heatmap of proportion of positive cells within segmented tumor cells from scRNA-seq with 12 different functional states of cancer cells and gene expression status by BD treated or not, Figure S5: Analysis of normal scRNA-seq data (GSM4909262) which is paired with GSM4909313 ER+ BC, Figure S6: Dot plots of expression of ligands receptor with percent of positively expressed and average of expression in ER+ sample GSM (GSM4909313), Table S1: Total genes for survival analysis with breast cancer patients from TCGA database, Table S2: Total genes for DEG analysis between pH 6.5 and pH 7.6, Table S3: Significant genes with prognosis and differential expressed between acidic (pH 6.5) and normal (pH 7.6) condition, Table S4: Significant genes with differential expressed between each compound treated and control group according to acidosis dependent prognosis-related genes, Table S5: Evaluation of effectiveness of compound to acidosis dependent prognosis-related manner, Table S6: Whole significantly differential expressed genes between BD treated and control group.
