# Supplementary material for: In Silico Evaluation of Natural Compounds for an Acidic Extracellular Environment in Human Breast Cancer
Source: Cells. 2021 Oct 6;10(10):2673. doi: 10.3390/cells10102673 (PMC8534855; doi:10.3390/cells10102673)
Supplement: Supplementary file 1 [file cells-10-02673-s001.zip › Supplementary Figures.pdf]

Supplementary Figures

A

GSM4909307 (ER+)

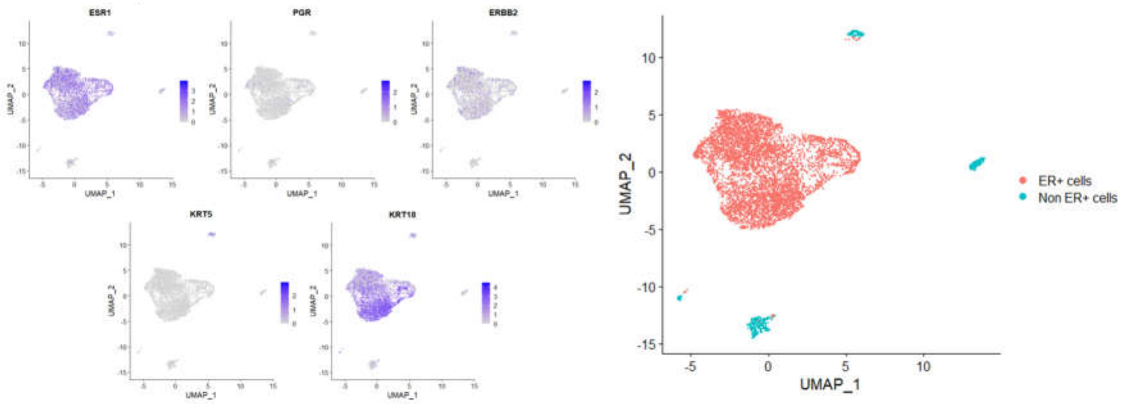

B

GSM4909299 (ER+)

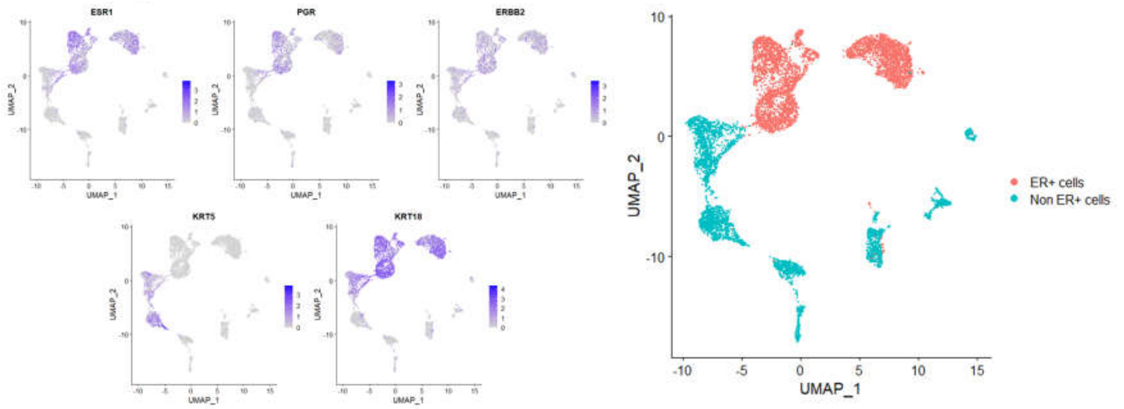

C

GSM4909315 (ER+)

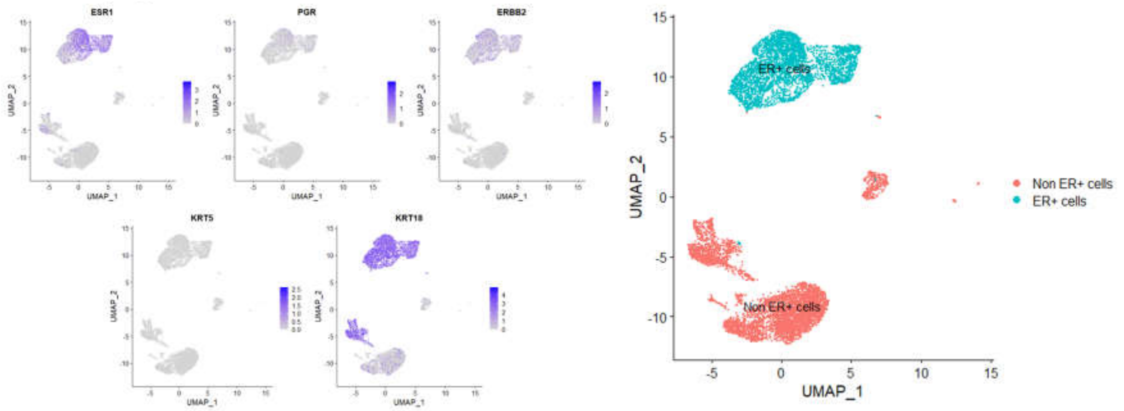

Figure S1 Segmentation of ER⁺ breast cancer cells from single cell data with patients.

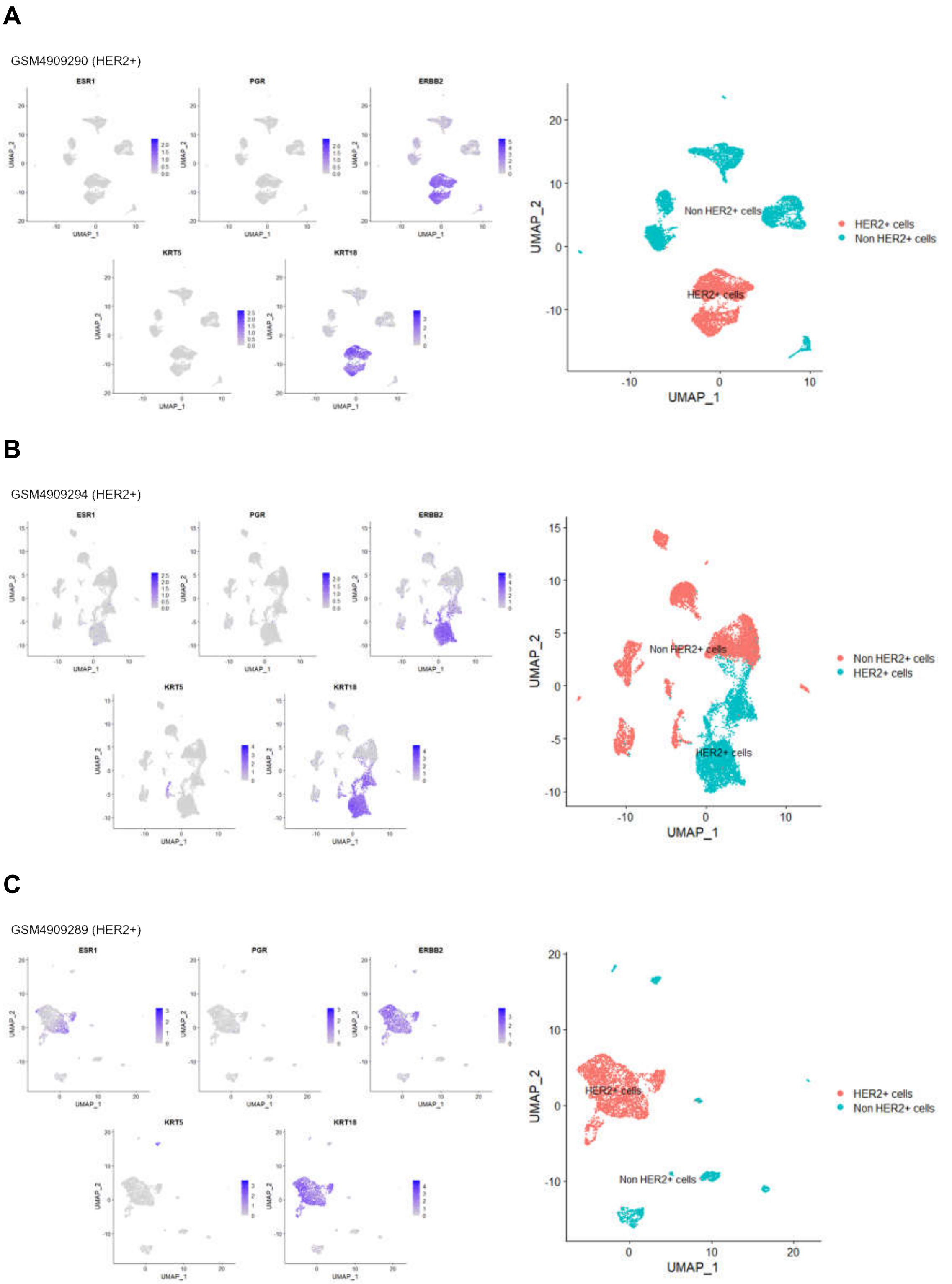

Figure S2 Segmentation of HER^{+} breast cancer cells from single cell data with patients.

A

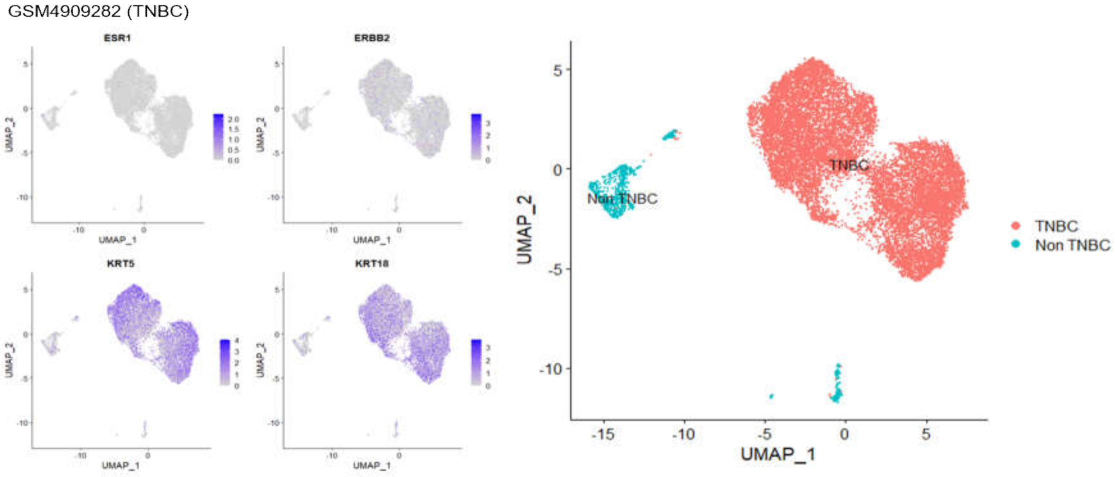

B

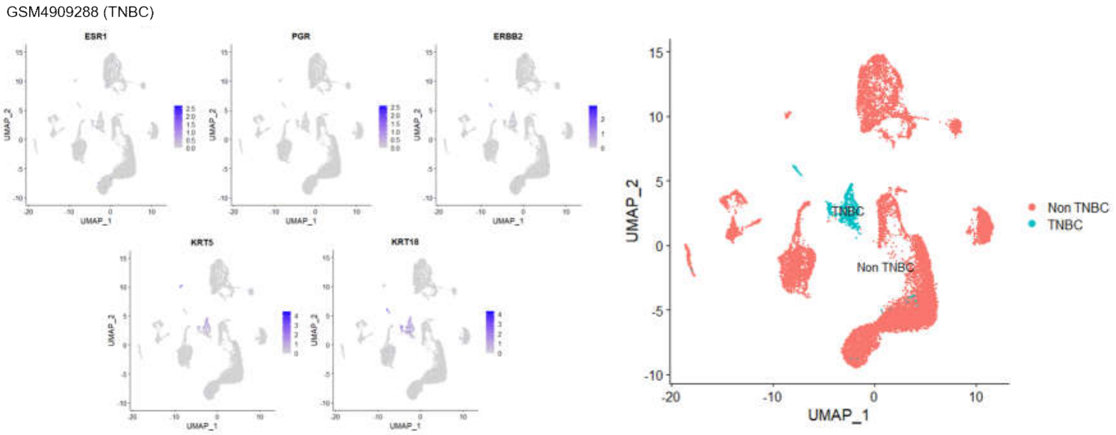

C

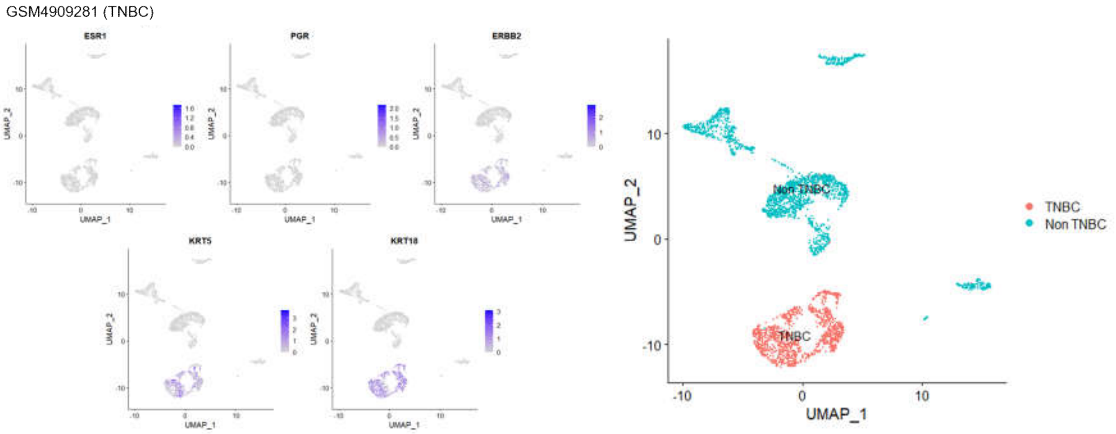

Figure S3 Segmentation of TN breast cancer cells from single cell data with patients.

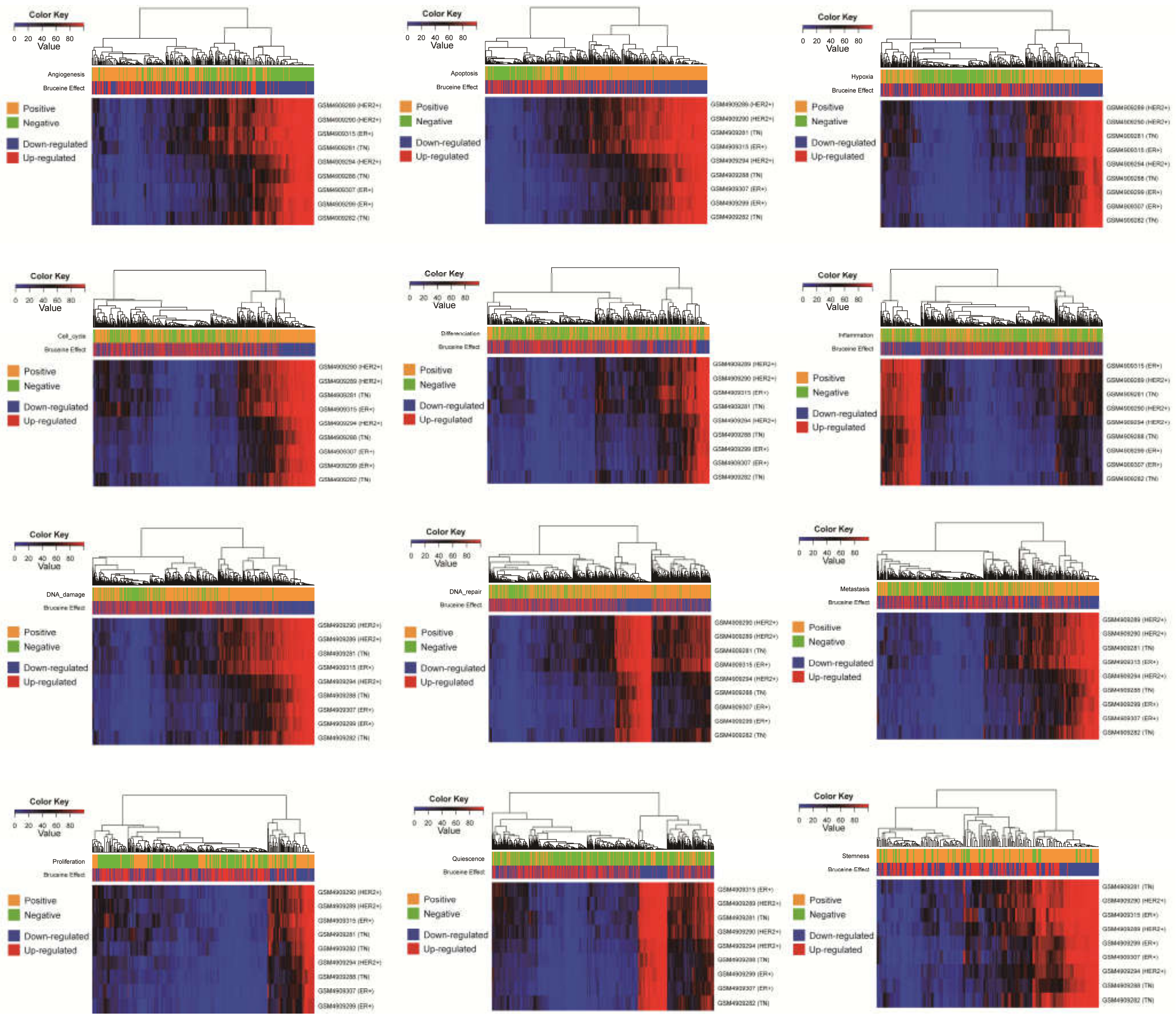

Figure S4 Heatmap of proportion of positive cells within segmented tumor cells from scRNA-seq with 12 different functional states of cancer cells and gene expression status by BD treated or not.

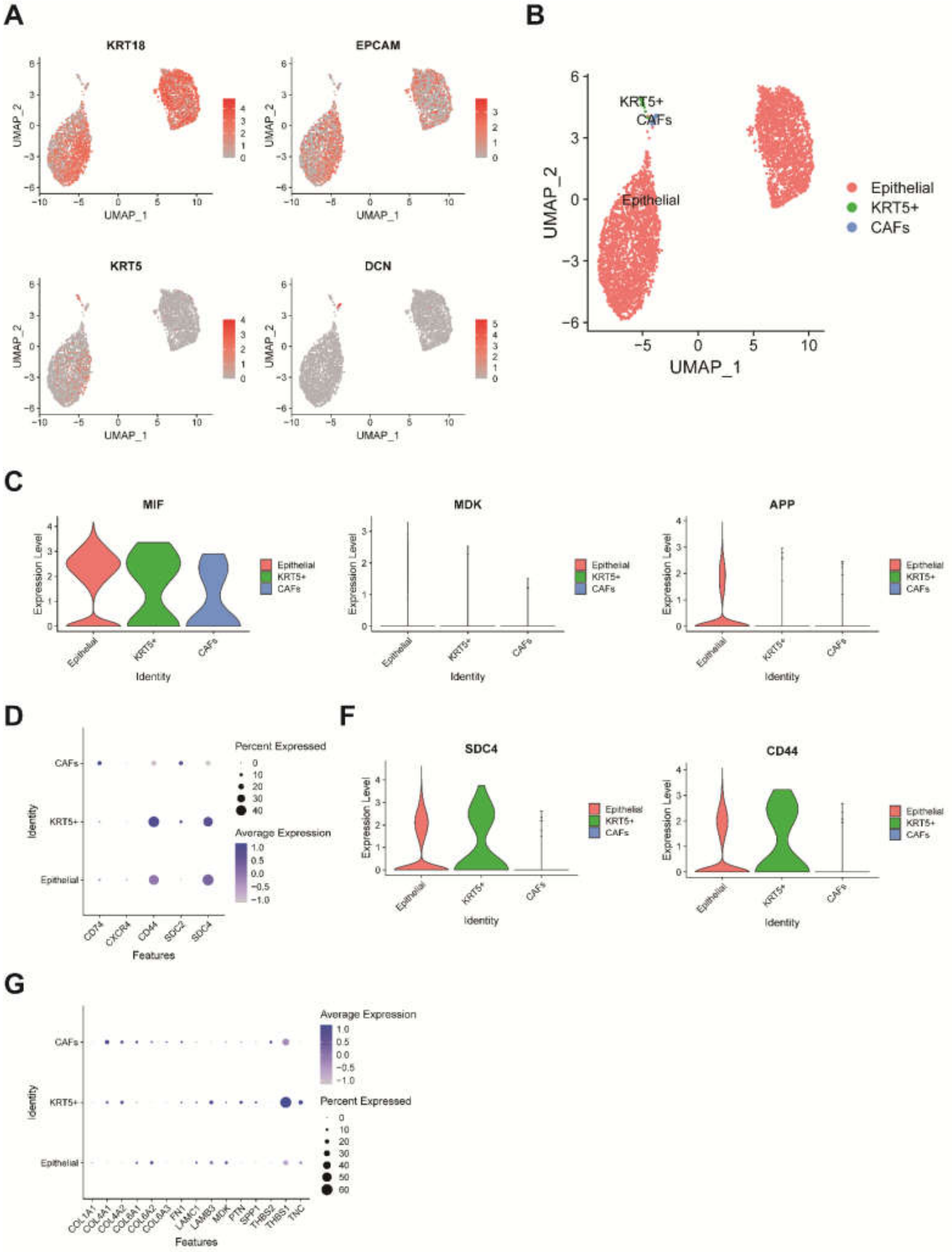

Figure S5 Analysis of normal scRNA-seq data (GSM4909262) which is paired with GSM4909313 ER$^+$ BC. (A) Feature plot of the specific markers (KRT18: Luminal, EPCAM: Epithelial, KRT5: basal-like, and DCN: Fibroblast), (B) Feature plot with cluster of cell types according to the specific markers, (C) Violin plot of ligands which were expressed in ER$^+$ sample GSM (GSM4909313), (D) Dot plot with percent of positively expressed and average of expression of ligands which were expressed in ER$^+$ sample GSM (GSM4909313), (E) Violin plot of receptors which were expressed in ER$^+$ sample GSM (GSM4909313) and (G) Dot plot with percent of positively expressed and average of expression of receptors which were expressed in ER$^+$ sample GSM (GSM4909313).

A

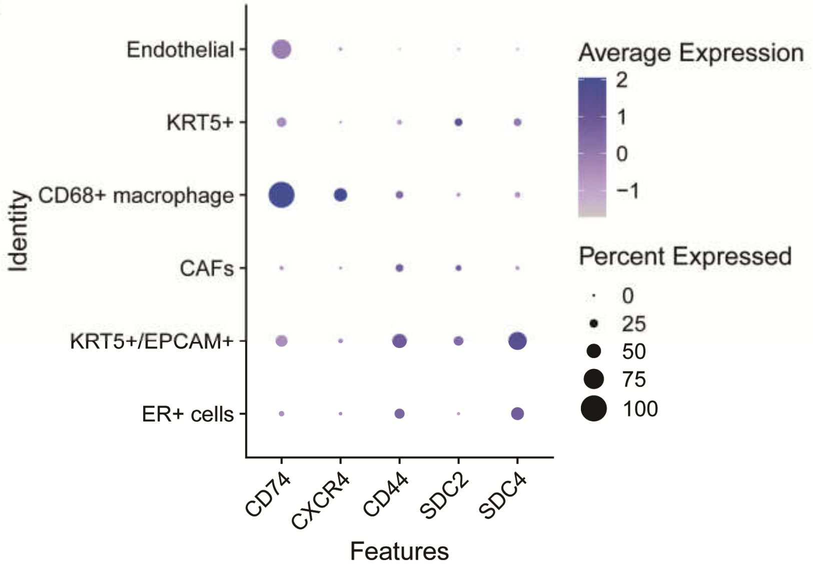

B

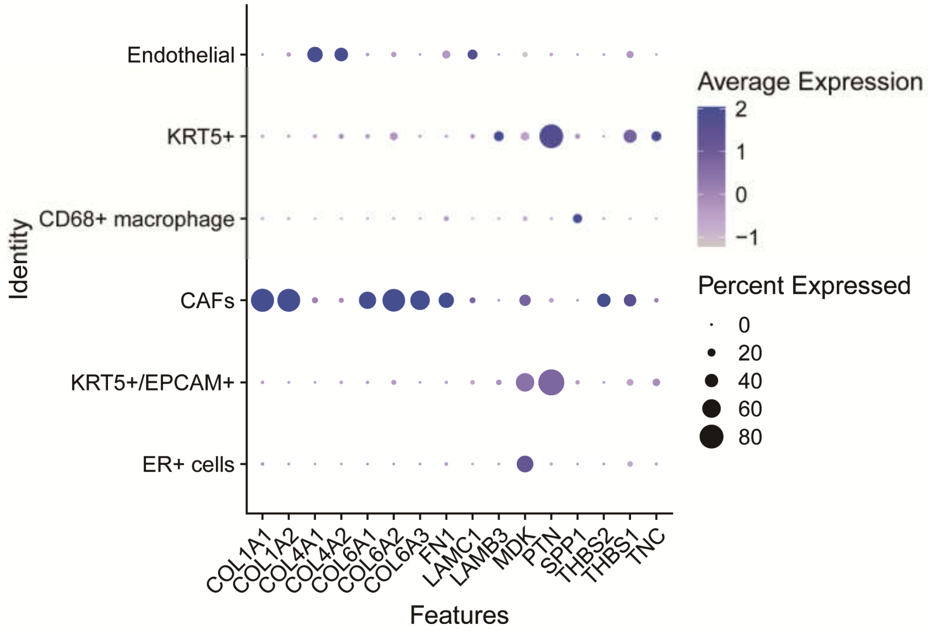

Figure S6 Dot plots of expression of ligands receptor with percent of positively expressed and average of expression in ER^+ sample GSM (GSM4909313). (A) dot plot of significant ligands from cell-to-cell interaction analysis and (B) dot plot of significant receptors from cell-to-cell interaction analysis.
